# Vaccination Readiness and Conspiracy Beliefs to Virus‐Based Diseases: A Validation Study

**DOI:** 10.1155/cjid/9100219

**Published:** 2026-08-10

**Authors:** Daniel Kwasi Ahorsu, Mei-Dan Lai, Jiajia Ye, Mark D. Griffiths, Servet Üztemur, Carol Strong, Nai-Ying Ko, Po-Ching Huang, Chung-Ying Lin

**Affiliations:** ^1^ Department of Special Education and Counselling, The Education University of Hong Kong, 10 Lo Ping Rd. Ting Kok New Territories, Hong Kong, China, ied.edu.hk; ^2^ Department of Public Health, College of Medicine, National Cheng Kung University, 1 University Rd., Tainan 701401, Taiwan, ncku.edu.tw; ^3^ Department of Rehabilitation Assessments, Rehabilitation Hospital Affiliated to Fujian University of Traditional Chinese Medicine, 53 North Wu Si Rd., Fuzhou 350003, China, fjtcm.edu.cn; ^4^ Psychology Department, Nottingham Trent University, 50 Shakespeare St, Nottingham NG1 4FQ, UK, ntu.ac.uk; ^5^ Department of Turkish and Social Sciences Education, Faculty of Education, Anadolu University, Yeşiltepe Yunus Emre Kampüsü Anadolu University, Eskişehir 26470, Türkiye, anadolu.edu.tr; ^6^ Department of Nursing, College of Medicine, National Cheng Kung University, 1 University Rd., Tainan 701401, Taiwan, ncku.edu.tw; ^7^ Department of Physiotherapy, School of Nursing and Health Sciences, Hong Kong Metropolitan University, 1 Sheung Shing St. Homantin Kowloon, Hong Kong, China, hkmu.edu.hk; ^8^ Institute of Allied Health Sciences, College of Medicine, National Cheng Kung University, 1 University Rd., Tainan 701401, Taiwan, ncku.edu.tw; ^9^ Biostatistics Consulting Center, National Cheng Kung University Hospital, College of Medicine, National Cheng Kung University, 1 University Rd., Tainan 701401, Taiwan, ncku.edu.tw; ^10^ Department of Occupational Therapy, College of Medicine, National Cheng Kung University, 1 University Rd., Tainan 701401, Taiwan, ncku.edu.tw; ^11^ School of Nursing, College of Nursing, Kaohsiung Medical University, 100 Shiquan 1st Rd., Kaohsiung 807378, Taiwan, kmu.edu.tw

**Keywords:** COVID-19 Conspiracy Beliefs Scale, COVID-19 Vaccination Readiness Scale, Influenza Conspiracy Beliefs Scale, Influenza Vaccination Readiness Scale, Taiwanese

## Abstract

Individuals’ conspiracy beliefs can influence their vaccination readiness, which may affect the annual vaccination uptake rate for infectious diseases such as influenza and COVID‐19. To provide reliable and valid tools adapted to the Taiwanese context in assessing these two constructs, the present study validated the psychometric properties of four scales among Taiwanese adults: the COVID‐19 Vaccination Readiness Scale (CVRS), Influenza Vaccination Readiness Scale (IVRS), Influenza Conspiracy Beliefs Scale (ICBS), and COVID‐19 Conspiracy Beliefs Scale (CCBS). A total of 914 participants completed the online survey between October and November, 2024. Confirmatory factor analysis (CFA) was employed to confirm the model structures and factor loadings for the four scales. All fit indices for all the scales were acceptable, with CVRS and IVRS fitting well to a bifactor model—general and specific—(comparative fix index [CFI] = 0.936 and 0.943) and the CCBS and ICBS to a two‐factor model (CFI = 0.961 and 0.973). The internal consistency assessed by Cronbach’s alpha [*α*] and McDonald’s omega [*ω*] was good among all scales (*α* = 0.87, 0.87, 0.93 and 0.96; *ω* = 0.90, 0.91, 0.95 and 0.97, for CVRS, IVRS, CCBS and ICBS). Moreover, conspiracy beliefs negatively correlated with vaccination readiness (*r* = −0.351 to −0.417, *p* < 0.01). The robustness of these scales was validated. Moreover, conspiracy beliefs were associated with vaccination readiness. Authorities may use these scales to understand individuals’ thoughts on vaccination and inform promotion strategies. Future studies may consider these variables in other types of infectious diseases, such as Mpox.

**Summary**
• Four scales assessing vaccination readiness and conspiracy beliefs for COVID‐19 and influenza were validated.
• Psychometric properties, including factorial validity, internal consistency, and construct validity, were well supported.
• Conspiracy beliefs were found to be negatively associated with vaccination readiness.

## 1. Introduction

Influenza, also known as flu, is a contagious viral infection with influenza types A and B being the primary causes of seasonal flu among humans. The transmission medium of influenza is through contact with respiratory secretions (i.e., large particle droplets of a sneeze and/or cough) of infected individuals. As an acute respiratory disease, it mainly affects respiratory organs and may also involve other organs such as the brain, heart, and muscles. Seasonal outbreaks of influenza usually occur in the winter but can evolve into an epidemic or pandemic (i.e., based on the number of cases, transmission and/or coverage area, etc.), causing significant morbidity and mortality worldwide [1, 2]. It is reported that globally, flu kills an average of 7 million individuals each year with the majority in South‐East Asia followed by Africa [3]. Given that the best preventive strategy is through vaccinations [1], the influenza vaccination has been offered as an annual public health policy in Taiwan [4].

The coronavirus disease 2019 (COVID‐19) is one of the latest respiratory diseases that shares similar characteristics with the flu. These common characteristics include both being caused by respiratory viruses, transmitted via respiratory droplets, aerosols, and contact with contaminated surfaces, manifesting symptoms (e.g., fever, cough, fatigue, sore throat, and body aches), potential for severe illness (e.g., pneumonia, hospitalization, and death), global spread (i.e., how it affects individuals worldwide), and preventable by vaccines [5–7]. Although COVID‐19 shares similar characteristics (e.g., signs and symptoms) with influenza, their contextual background is different. The outbreak of COVID‐19 was sudden and transmitted by a more infectious virus, with longer illness duration, a broader range of severe complications, and more serious comorbid conditions, thereby posing a higher level of threat to vulnerable populations such as older adults or children with immature immune systems [8–10]. By November 2025, COVID‐19 had affected over 778 million individuals with over 7 million deaths worldwide [11] and the number is still increasing, making it the most pervasive respiratory disease globally. It is also agreed that COVID‐19 vaccination is the most effective way to prevent COVID‐19, with continued vaccination being provided by the Taiwan government [12].

Given the contextual differences between influenza and COVID‐19, having different scales for each will help identify different vaccination strategies, thus informing their individual management strategies. However, before vaccination uptake can be successful, individuals must know, understand, and trust the essence of the vaccine. That is, there seems to be combination of factors that influence an individual’s decision to get vaccinated. Previous studies have examined aspects of vaccination uptake and have developed the 7C model (i.e., confidence, complacency, constraints, calculation, collective responsibility, compliance, and conspiracy) which underpins vaccination readiness (i.e., a set of components that increases or decreases the likelihood an individual getting vaccinated) [13, 14]. In addition to the 7C model, there is the 3C model (confidence, complacency, and convenience) and the 5C model (confidence, complacency, constraints, calculation, and collective responsibility) which share similar components. The 7C model was preferred to the other two models because it is an extension of the 3C and 5C models, and more comprehensively covers a wider area of vaccination readiness [13]. Moreover, the inclusion of constructs such as “conspiracy” and “compliance” makes it particularly suited to address the complexities of vaccine hesitancy in today’s information landscape compared to other models. Although general health behavior models such as the Health Belief Model and the Theory of Planned Behavior have been widely applied [15, 16], the present study adopted the 7C model because it offers a vaccination‐specific framework that directly addresses key psychological antecedents of vaccination readiness.

Individuals’ trust in the safety and effectiveness of vaccines and health authorities (i.e., confidence) can be important to vaccination behavior. During the COVID‐19 pandemic, it was found that, in the different contributions, trust in the COVID‐19 vaccine had the strongest correlation with vaccine acceptance [14, 17, 18]. Apart from trust, the perceived risk of vaccine‐preventable diseases or vaccine adverse events (i.e., complacency) is considered a higher risk factor associated with vaccination behavior [19]. Individuals who think “the situation is not serious enough” and/or “I am not at risk” may be hesitant to vaccinate, while those who are chronically sick or pregnant may choose to vaccinate because they perceive themselves as at high risk [20]. In addition, when obstacles in daily life restrict vaccination (i.e., constraints), or when the personal costs exceed the benefits of receiving a vaccination (i.e., calculation), an individual may choose not to vaccinate [21]. Moreover, an individual’s willingness to protect others by getting vaccinated (i.e., collective responsibility), engaging in acts that support social monitoring and sanctioning of individuals refusing vaccination (i.e., compliance), and lower belief in vaccination‐related conspiracy theories and fake news (i.e., conspiracy) [13, 14] may also influence vaccination uptake.

One of the major barriers to vaccination campaigns involves individuals harboring the idea that there is a sinister ulterior motive behind the vaccination (i.e., conspiracy), which is reinforced by fake news regarding the vaccine. The belief in vaccination‐related conspiracy theories and fake news was significantly observed and reported during the COVID‐19 pandemic and resulted from convergence of multiple structural factors [22, 23]. The conspiracy became particularly widespread because the vaccines were introduced rapidly during a highly uncertain and fear‐driven pandemic and were further amplified by the rapid spread of fake news and emotionally driven misinformation on social media. Consequently, it substantially inhibited vaccination campaigns and acceptance rates [24–26].

Unfortunately, the belief in vaccination‐related conspiracy theories and fake news cut across health and non‐health professionals, as well as young and old individuals, which indicates its ubiquitous nature and potential threat during future influenza and COVID‐19 outbreaks [27]. Consequently, there is a need to assess such tendencies in order to develop strategies to improve vaccination uptake. The 7C model provides the key areas for assessing the readiness of an individual for vaccination uptake. Conspiracy thinking and beliefs are of interest here because they have the capacity to disrupt the management of infectious diseases [14, 17, 18]. Therefore, a psychometrically validated instrument to assess the 7Cs of vaccination readiness and vaccination conspiracy is needed for health authorities to assess the individuals’ attitude toward vaccination uptake within Taiwan’s unique cultural and sociopolitical environment.

The situation surrounding viral infections (e.g., influenza) and their vaccination among Taiwanese is not significantly different from those seen globally. The 1918 H1N1 Spanish flu pandemic was the earliest recorded influenza in Taiwan followed by several others including the 1957 H2N2, 1968 H3N2, 2003 severe acute respiratory syndrome (SARS), and the 2009 new H1N1 [28]. With the implementation of disease prevention fund held by the Taiwan Centers for Disease Control, the seasonal influenza vaccination has been provided annually since 1998. Comprehensive strategies have been adopted to tackle the specific influenza types based on past data [4, 9, 10, 28]. Moreover, the government has used different strategies (e.g., sending flyers, cooperating with primary healthcare clinics) to inform individuals about who is eligible for vaccination. At present, both seasonal influenza and COVID‐19 vaccinations are regularly provided in Taiwan’s annual vaccination project. Although vaccination is the most effective method for prevention and control of influenza and COVID‐19, it is getting more challenging to promote vaccination because vaccine hesitancy grew more prominently during the COVID‐19 pandemic due to the spread of misinformation [29]. Consequently, there is a need to develop ways of assessing the readiness and conspiracy beliefs of Taiwanese individuals to vaccination uptake, which would be beneficial in enhancing vaccination campaigns and health in general.

Although several scales assessing vaccination readiness and conspiracy beliefs have been developed, these mostly focus on general vaccination [13, 30]. Therefore, the present study aimed to validate the psychometric properties of four scales: the COVID‐19 Vaccination Readiness Scale (CVRS), Influenza Vaccination Readiness Scale (IVRS), Influenza Conspiracy Beliefs Scale (ICBS), and COVID‐19 Conspiracy Beliefs Scale (CCBS) among Taiwanese adults. Informed by the 7C framework, the present study developed parallel, vaccine‐specific measures of vaccination readiness and conspiracy beliefs for COVID‐19 and influenza. This approach allows for direct cross‐vaccine comparisons and provides a more fine‐grained assessment of vaccine‐related psychological processes within a specified cultural context. These validated scales may be useful for public health research and practice by helping to identify vaccine‐specific barriers, such as low readiness or conspiracy beliefs, and to inform more targeted vaccination interventions.

## 2. Methods

### 2.1. Participants and Procedures

A cross‐sectional study employing convenience and snowball sampling methods was conducted to collect the data. Because individuals who are aged over 18 years are considered legal adults in Taiwan who can decide their own vaccination willingness, the inclusion criteria were (i) being aged 18 years and older, (ii) currently living in Taiwan, (iii) being able to read and understand Chinese, and (iv) willing to provide consent to participate. Individuals who were advised by doctors not to have influenza or COVID‐19 vaccination (e.g., had previous severe allergy history) were excluded. The authors used their personal contacts of individuals residing in Taiwan to help distribute the online survey using *SurveyMonkey* between October and November, 2024. October is the time of year that the Taiwan Government publicize their annual vaccination campaign for COVID‐19 and influenza, and targets specific vulnerable populations (e.g., the elderly, children up to the age of 6 years, healthcare providers).

The study was conducted in accordance with the Declaration of Helsinki. The consent form was presented on the first page of the online survey. By clicking “yes,” the participants gave their consent to participate and proceeded to the survey questions. By clicking “no,” the survey ended directly and could not be further accessed. Participants who completed the survey received 100 New Taiwan Dollars (approximately 3.3 US dollars) as compensation. All participants provided written informed consent prior to participating in the study. The study protocol was approved by the National Cheng Kung University Human Research Ethics Committee (approval no: NCKU HREC‐E‐113‐621‐2).

### 2.2. Scales Development and Translation Procedures

The CVRS and the IVRS were developed based on the 7C Vaccination Readiness Scale [13] by replacing the word “vaccines” with “COVID‐19 vaccination” and “influenza vaccination,” respectively. The ICBS was developed based on the CCBS [27] by replacing the word “COVID‐19” with “influenza.”

All four scales were then translated into Traditional Chinese following standard translation procedures. Forward translation was conducted by four translators composed of three with vaccination‐related backgrounds and one without. Backward translation was conducted by two translators who were fluent in English and had no medical background. A multidisciplinary expert panel then reviewed and compared the original and translated versions. Necessary revisions were made to ensure consistency between the two versions. Twenty‐two participants with occupations in medical, health, and educational sectors, as well as full‐time students, were invited to complete a pilot test. Based on their feedback, further wording adjustments were made to finalize the scales.

### 2.3. Measures

#### 2.3.1. CVRS

The CVRS is a 21‐item scale used to assess an individual’s readiness for receiving COVID‐19 vaccine. The scale is based on the 7C scale of vaccination readiness [13]. The 7Cs are made up of confidence, complacency, constraints, calculation, collective responsibility, compliance, and conspiracy. The CVRS has three items for each of the C variables, totaling 21 items. Each of the items is rated on a seven‐point Likert scale from 1 (strongly disagree) to 7 (strongly agree). Items 4, 5, 9, 10, 11, 12, 19, 20, and 21 are reverse‐coded. A higher score indicates a greater level of vaccination readiness. All the items were translated into Traditional Chinese language for Taiwanese. An example item is “COVID‐19 vaccination side effects occur rarely and are not severe for me”. The scale’s psychometric properties are reported in the Results section.

#### 2.3.2. IVRS

The IVRS was developed to assess an individual’s readiness for receiving influenza vaccination based on the 7C scale of vaccination readiness [13]. The IVRS has three items per C variable (i.e., confidence, complacency, constraints, calculation, collective responsibility, compliance, and conspiracy), totaling 21 items. Each of the items is rated on a seven‐point Likert scale from 1 (strongly disagree) to 7 (strongly agree). Items 4, 5, 9, 10, 11, 12, 19, 20, and 21 are reverse‐coded. A higher score indicates a greater level of vaccination readiness. All the items were translated into Traditional Chinese language for Taiwanese. An example item is “Influenza vaccination side effects occur rarely and are not severe for me”. The scale’s psychometric properties are reported in the Results section.

#### 2.3.3. CCBS

The CCBS is a 13‐item scale used to assess an individual’s COVID‐19 conspiracy beliefs. The present study adapted the original scale [27] by translating it into Traditional Chinese language for Taiwanese. Items are rated on a five‐point Likert scale from 5 (strongly agree) to 1 (strongly disagree) with a total score between 13 and 65. A higher score indicates greater COVID‐19 conspiracy beliefs. An example item is “In my opinion, COVID‐19 does not exist”. The original scale had acceptable psychometric properties [27]. The scale’s psychometric properties are reported in the Results section.

#### 2.3.4. ICBS

The ICBS is an adapted scale based on the original CCBS, with “COVID‐19” replaced with “influenza.” The ICBS is used to assess an individual’s influenza conspiracy beliefs. The original CCBS is a 13‐item scale developed by Gökalp et al. [27]. The items were then translated into Traditional Chinese language for Taiwanese. Each item is rated on a five‐point Likert scale from 5 (strongly agree) to 1 (strongly disagree) with a total score between 13 and 65. A higher score indicates greater influenza conspiracy beliefs. An example item is “In my opinion, influenza does not exist.” The scale’s psychometric properties are reported in the Results section.

### 2.4. Data Analysis

Descriptive analysis was used to assess the participants’ baseline characteristics and confirmatory factor analyses (CFAs) were employed to confirm the model structure and factor loading for the four scales separately. For the CVRS and IVRS, a bifactor model (i.e., including a general readiness factor and seven specific readiness factors [confidence, complacency, constraints, calculation, collective responsibility, compliance, and conspiracy]) was examined based on the literature [13, 31–34]. For the CCBS and ICBS, a two‐factor structure (i.e., concealed facts and irrational suspicion factor, and global control factor) was examined based on the literature [27]. Similar to the analytic methods of previous studies [13, 31–34], the CFAs in the present study were analyzed using the maximum likelihood estimation with robust standard errors (MLR).

Four fit indices, including comparative fit index (CFI), Tucker‐Lewis index (TLI), root mean square error of approximation (RMSEA) and standardized root mean square residual (SRMR), were used to examine the model fitness. Regarding the recommended cut‐offs indicating good fit, CFI and TLI should be greater than 0.90 [35], and RMSEA and SRMR should be less than 0.08 [35, 36]. After confirming the factor structure for the four measures, Cronbach’s alpha value [*α*] and McDonald’s omega value [*ω*] were used for assessing the internal consistency for each scale. All analyses were performed using *R* software: CFAs by the *lavaan* package and internal consistency by the *psych* package [37, 38].

## 3. Results

A total of 914 participants responded to the survey with a greater proportion being female (58.6%), aged 50–59 years (28.3%), working in the commerce and industry sector (30.9%), having an undergraduate level of education (50.1%), and living in the northern part of Taiwan (42.7%). In the year 2023–2024, 507 participants received the influenza vaccination (55.5%) while 515 received the COVID‐19 vaccination (56.3%), with further details reported in Table 1.

**TABLE 1:** Baseline demographics of participants (*n* = 914).

Variable	*n* (%)
Sex	
Female	536 (58.6)
Male	378 (41.4)
Age (in years)	
18–29	173 (18.9)
30–39	208 (22.8)
40–49	138 (15.1)
50–59	259 (28.3)
60–64	79 (8.6)
Over 65	57 (6.2)
Job	
Student	47 (5.1)
Health‐related	152 (16.7)
Education‐related	88 (9.6)
Commerce and industry	282 (30.9)
Service Sector	154 (16.9)
Homemaker	58 (6.4)
Retired	63 (6.9)
Others	70 (7.7)
Education	
Junior high school or below	28 (3.1)
Senior high school or vocational school	79 (8.6)
Associate degree	101 (11.1)
Undergraduate	465 (50.1)
Graduate or above	241 (26.4)
Residence	
Northern Taiwan	390 (42.7)
Central Taiwan	158 (17.3)
Southern Taiwan	331 (36.2)
Eastern Taiwan and outlying islands	35 (3.8)
Received influenza vaccination in the past 1 year	
Yes	507 (55.5)
No/unsure	407 (44.5)
Received COVID‐19 booster dose in the past 1 year	
Yes	515 (56.3)
No/unsure	399 (43.7)

Table 2 shows the CFA fit indices of four measures (i.e., CVRS, IVRS, CCBS, and ICBS). All the CFA fit indices were acceptable, indicating that the CVRS and IVRS fitted well with a bifactor model—general and specific—(CFI = 0.936 and 0.943, TLI = 0.903 and 0.914, RMSEA = 0.065 and 0.066, and SRMR = 0.043 and 0.040) and that the CCBS and ICBS fitted well with a two‐factor model (CFI = 0.961 and 0.973, TLI = 0.949 and 0.965, RMSEA = 0.075 and 0.074, and SRMR = 0.043 and 0.035) (see Table 2).

**TABLE 2:** Fit indices of four measures in confirmatory factor analysis.

	CVRS	IVRS	CCBS	ICBS
*CFA*				

*χ* ^2^ (df)	679.316 (139)	698.815 (140)	365.493 (59)	363.987 (60)
*p* value	< 0.001	< 0.001	< 0.001	< 0.001
CFI	0.936	0.943	0.961	0.973
TLI	0.903	0.914	0.949	0.965
RMSEA	0.065	0.066	0.075	0.074
SRMR	0.043	0.040	0.043	0.035

*Note:* SRMR, standardized root mean square residual.

Abbreviations: CCBS, the COVID‐19 Conspiracy Beliefs Scale; CFA, confirmatory factor analysis; CFI, comparative fit index; CVRS, the COVID‐19 Vaccination Readiness Scale; ICBS, the Influenza Conspiracy Beliefs Scale; IVRS, the Influenza Vaccination Readiness Scale; RMSEA, root mean square error of approximation; TLI, Tucker‐Lewis index.

The CFA factor loadings of the items of each scale are presented in Supporting Table S1 (i.e., CVRS and IVRS) and Table S2 (i.e., CCBS and ICBS). For CVRS and IVRS, each item had two factor loadings: one factor loading from the general factor (i.e., overall vaccination readiness) and another from a specific factor (i.e., a specific vaccination readiness). When scrutinizing factor loadings in the general factor, some items had a loading issue (i.e., < 0.3), including Item 4 (loading = 0.167), Item 5 (0.282), Item 9 (0.192), Item 10 (−0.111), Item 11 (−0.085), and Item 12 (−0.151) in the CVRS; and Item 4 (0.134), Item 5 (0.186), Item 10 (−0.232), Item 11 (−0.231), Item 16 (0.079), and Item 18 (0.082) in the IVRS. Regarding factor loadings in the specific factor for the CVRS and IVRS, most were 0.3 or above, except for Item 6 (−0.176) and Item 9 (−0.159) for the CVRS; and Item 6 (−0.077) and Item 17 (0.257) for the IVRS (Supporting Table S3). For the CCBS and ICBS, both measures showed good factor loadings (i.e., > 0.45) for all items (Supporting Table S4).

Table 3 shows the internal consistencies (i.e., Cronbach’s *α* and McDonald’s *ω*) of the four measures (i.e., CVRS, IVRS, CCBS, and ICBS) across their subscales. The overall McDonald’s *ω* for the CVRS and IVRS were 0.90 and 0.91, respectively, and their subscales ranged between 0.70 and 0.84, and 0.74 and 0.86, respectively. The overall Cronbach’s *α* for the CVRS was 0.87, and the subscales were between 0.72 and 0.84, except for constraints (*α* = 0.55) and calculation (*α* = 0.69). The overall Cronbach’s *α* was 0.87 for the IVRS, and the subscales were between 0.73 and 0.86, except for constraints (*α* = 0.64). The overall McDonald’s *ω* for the CCBS and ICBS were 0.95 and 0.97, respectively, and their subscales ranged between 0.93 and 0.94, and 0.95 and 0.96, respectively. The overall Cronbach’s *α* for the CCBS and ICBS were 0.93 and 0.96, respectively, and that of their subscales ranged between 0.89 and 0.91, and 0.93 and 0.94, respectively.

**TABLE 3:** Internal consistency of four measures.

	Cronbach’s α	McDonald’s ω	Cronbach’s α	McDonald’s ω
	CVRS		IVRS	
Overall	0.87	0.90	0.87	0.91
Confidence	0.72	0.76	0.73	0.77
Complacency	0.79	0.83	0.82	0.85
Constraints	0.55	0.70	0.64	0.74
Calculation	0.69	0.72	0.74	0.79
Collective responsibility	0.84	0.84	0.86	0.86
Compliance	0.74	0.75	0.75	0.79
Conspiracy	0.72	0.72	0.76	0.76
	CCBS		ICBS	
Overall	0.93	0.95	0.96	0.97
Concealed facts and irrational suspicion	0.91	0.94	0.93	0.95
Global control	0.89	0.93	0.94	0.96

Abbreviations: CCBS, the COVID‐19 Conspiracy Beliefs Scale; CVRS, the COVID‐19 Vaccination Readiness Scale; ICBS, the Influenza Conspiracy Beliefs Scale; IVRS, the Influenza Vaccination Readiness Scale.

Table 4 shows the correlations between the four vaccine attitude measures. There were significant and positive relationships between scores on the IVRS and CVRS (*r* = 0.73, *p* < 0.001), and the ICBS and CCBS (*r* = 0.84, *p* < 0.001). Also, there were significant and negative relationships between scores on the CCBS and CVRS (*r* = −0.42, *p* < 0.001), ICBS and CVRS (*r* = −0.35, *p* < 0.001), IVRS and CCBS (*r* = 0‐0.35, *p* < 0.001), and ICBS and IVRS (*r* = −0.35, *p* < 0.001).

**TABLE 4:** Correlations among four vaccine attitude measures (*n* = 914).

	CVRS	CCBS	IVRS	ICBS
CVRS	—			
CCBS	−0.417	—		
IVRS	0.732	−0.354	—	
ICBS	−0.351	0.836	−0.354	—

*Note:* All *p* values < 0.001.

Abbreviations: CCBS, the COVID‐19 Conspiracy Beliefs Scale; CVRS, the COVID‐19 Vaccination Readiness Scale; ICBS, the Influenza Conspiracy Beliefs Scale; IVRS, the Influenza Vaccination Readiness Scale.

## 4. Discussion

The present study validated and evaluated the psychometric properties of the CVRS, IVRS, CCBS, and ICBS among Taiwanese adults. The results showed that, in general, the four scales had robust psychometric properties. With regard to the CVRS and IVRS, the results fitted well with a bifactor model—general and specific—similar to previous studies [32, 34]. That is, the CFA fits and internal reliability coefficients were all acceptable. The novel aspect of the present study was that both CVRS and IVRS were developed and examined separately unlike most previous studies that examined the psychometric properties of a vaccination readiness scale, sometimes in reference to COVID‐19 [13, 31–34].

The results also showed that there was a positive relationship between the CVRS and the IVRS, indicating the validity of the IVRS, especially because most previous studies used the readiness scale in the context of COVID‐19 [13, 31–34]. These findings indicate that both readiness scales are suitable to be used among Taiwanese adults to examine their readiness for COVID‐19 and influenza vaccinations.

Moreover, the results on the CFA fit indices showed that the indices fitted well to a two‐factor model for both conspiracy beliefs scales (i.e., ICBS and CCBS), and the CFA fit indices were generally acceptable as well as their internal reliability coefficients. Conspiracy theories surrounding a disease and/or its treatment/prevention are mostly inimical to the health of individuals and public health in general, as is the case with viral infectious diseases such as COVID‐19 and SARS. Consequently, serious efforts must be made to provide accurate information to minimize conspiracy beliefs before they harm the public. One way to objectively ascertain this is through assessment which makes the present study vital to future viral infection‐related pandemics. Importantly, the present study developed the ICBS based on the CCBS [27], which expands the extent to which health experts can assess conspiracies. The possible detrimental effects of influenza and COVID‐19 conspiracy beliefs on vaccination readiness are manifested in the relationship between these variables in the present study.

There were negative associations between both conspiracy beliefs (i.e., CCBS and ICBS) and vaccination readiness (i.e., CVRS and IVRS), which suggests that individuals with high conspiracy beliefs tend to have lower vaccination readiness. However, there was a positive association between the ICBS and CCBS, indicating their suitability for examining conspiracy beliefs. This finding is similar to previous studies, which have examined conspiracy beliefs and COVID‐19 vaccination [39, 40]. Although the correlation coefficients were relatively high between CVRS and IVRS (*r* = 0.732) and between CCBS and ICBS (*r* = 0.836), the literature posits that a correlation coefficient < 0.85 indicates two separate constructs. Therefore, the CVRS, IVRS, CCBS, and ICBS were considered four different scales based on psychometric guidelines of previous studies [41–43].

Although several items in the CVRS and IVRS showed low loadings on the general factor, this finding should be interpreted cautiously. Within a bifactor framework, such a pattern suggests that some items are more strongly associated with specific dimensions of vaccination readiness rather than the overarching construct. This interpretation is supported by the acceptable overall model fit indices observed for both scales. From a theoretical perspective, these items represent conceptually meaningful components of vaccination readiness that are essential for maintaining adequate content coverage. Therefore, they were retained to avoid construct underrepresentation and to preserve the multidimensional nature of the scales. However, future validation studies may consider monitoring or refining these items across different populations and cultural settings to further improve their psychometric performance. Moreover, the constraints‐related subscales of the CVRS and IVRS showed comparatively modest reliability estimates relative to the other constructs assessed. Therefore, findings derived from these subscales should be interpreted with caution, particularly when used independently. However, these subscales may still provide useful information regarding perceived barriers to vaccination and contribute to a more comprehensive understanding of vaccination readiness.

Overall, conspiracy beliefs (i.e., CCBS and ICBS) and vaccination readiness (i.e., CVRS and IVRS) may be considered valid instruments for use in vaccination programs. However, because convenience and snowball sampling were used in the present study, the sample may have disproportionally included individuals with greater interest in health‐related topics or greater social media engagement. Consequently, the levels of conspiracy beliefs and vaccination readiness observed in the study may not fully reflect those of the general Taiwanese population, thereby limiting the external validity and generalizability of the findings. Future studies using probability‐based sampling methods are warranted to improve population representativeness and further validate the present findings.

### 4.1. Strengths and Limitations

The strengths of the present study include the development and translation of four scales (i.e., CVRS, IVRS, CCBS, and ICBS) for Taiwanese adults. These will help health experts to objectively assess the vaccination readiness and conspiracy beliefs among citizens. Moreover, these four scales will be useful for the Taiwan government to monitor or promote vaccination campaigns. Subsequently, the Taiwan government may effectively achieve herd immunity during the peak season of COVID‐19/influenza. Practically, these scales can be used in public health studies (or during real‐life vaccination programs) to examine the effect of conspiracy theories or fake news in interfering with vaccination programs. These scales can also be integrated into public health surveys to help identify hesitant populations or inform targeted messaging strategies. Moreover, health authorities may adopt components from the 7C model while promoting general vaccination programs among adults. Additionally, the sample used in the present study was relatively large [44], and across a wide range of groups including different educational levels, jobs, sex, age, and places of residence.

The present study also has some limitations. First, the study included only adults (i.e., 18 years old and above) without details on how the CVRS, IVRS, CCBS, and ICBS can be applied to other vulnerable populations such as older adults, pregnant women, or children and adolescents. Future studies may examine the psychometric properties of these scales among other populations. Also, studies may examine the influence of vaccine readiness and conspiracy beliefs among carers of vulnerable populations (e.g., whether guardian’s/parent’s vaccination readiness and conspiracy beliefs may influence their offspring). Second, the present study focused on psychometric validation, and the sample was not intended to be population‐representative. Moreover, participants were recruited using convenience and snowball sampling methods, which may have disproportionately attracted individuals with greater interest in health‐related topics or higher engagement with social media. Consequently, the observed levels of vaccination readiness and conspiracy beliefs may not fully reflect those of the broader Taiwanese population. Therefore, caution is warranted when generalizing the findings beyond the study sample. Future research employing population‐representative samples is needed to further evaluate the external validity of the four measures. Third, the use of cross‐sectional design data may limit the extent of causality. Fourth, the self‐reported data may be affected by social desirability biases and/or poor memory recall. Future studies may examine the test–retest reliability of these scales as they may be used repeatedly among the citizens. Fifth, although the overall model fit was acceptable, some items in the CVRS and IVRS showed relatively low loadings on the general factor. Future research should further evaluate these items using alternative psychometric approaches (e.g., item response theory) and consider scale refinement. Lastly, although the present findings support the psychometric properties of the scales (i.e., CVRS, IVRS, CCBS, and ICBS) and demonstrate associations between vaccination readiness and conspiracy beliefs, the study did not evaluate actual vaccination behavior, vaccine uptake, or longitudinal changes in attitudes. Therefore, future studies may examine the relationship between these scales and real‐world vaccination outcomes because this would further strengthen their practical utility and public health relevance. More specifically, evidence from longitudinal studies is needed to examine whether the relationships between conspiracy beliefs and vaccination readiness are bidirectional, and whether the relationships are influenced by other variables not collected in the present study (e.g., social, educational, or cultural factors).

## 5. Conclusion

Because vaccination readiness and conspiracy belief may be associated with vaccine uptake, and due to the lack of specific assessment instruments, the present study developed four psychometric scales (i.e., CVRS, IVRS, CCBS, and ICBS) to assess these two constructs in relation to vaccines for influenza and COVID‐19. Their psychometric properties were validated among Taiwanese adults. The CFA fit indices for all the scales were acceptable indicating their suitability in assessing the vaccination readiness and conspiracy beliefs among Taiwanese adults. The negative association between vaccination readiness and conspiracy beliefs suggests that health experts and health authorities should be forthright and trustworthy during their communication about respiratory diseases to the public, especially toward the general adult population. Moreover, components from the 7C model can be adopted when promoting vaccination to the adult population. However, for other vulnerable populations, such as older people, pregnant women, or children and adolescents, further investigations are warranted. The influence of carers on vulnerable populations can also be investigated to inform more comprehensive approach strategies to promote vaccination among those with higher vulnerability. Lastly, future studies may consider using the scale constructs to investigate vaccine readiness and conspiracy beliefs toward other types of infectious diseases, such as Mpox [45, 46].

## Author Contributions

Daniel Kwasi Ahorsu: conceptualization, methodology, validation, writing–original draft, and writing–review and editing.

Mei‐Dan Lai: conceptualization, data curation, formal analysis, methodology, resources, software, visualization, and writing–review and editing.

Jiajia Ye: methodology, validation, and writing–review and editing.

Mark D. Griffiths: methodology, validation, and writing–review and editing.

Servet Üztemur: conceptualization, investigation, resources, validation, and writing–review and editing.

Carol Strong: data curation, funding acquisition, investigation, resources, validation, and writing–review and editing.

Nai‐Ying Ko: data curation, funding acquisition, investigation, resources, validation, and writing–review and editing.

Po‐Ching Huang: conceptualization, data curation, funding acquisition, investigation, resources, validation, and writing–original draft.

Chung‐Ying Lin: conceptualization, data curation, funding acquisition, investigation, project administration, resources, supervision, writing–original draft, and writing–review and editing.

## Funding

This work was supported in part by (received funding from) the National Science and Technology Council, Taiwan under Grant [NSTC 114‐2321‐B‐006‐012].

## Conflicts of Interest

The authors declare no conflicts of interest.

## Supporting Information

Additional supporting information can be found online in the Supporting Information section.

## Supporting information


**Supporting Information** Table. S1. Factor loading of the COVID‐19 Vaccination Readiness Scale (CVRS) and the Influenza Vaccination Readiness Scale (IVRS) in confirmatory factor analysis. Table. S2. Factor loading of the COVID‐19 Conspiracy Beliefs Scale (CCBS) and the Influenza Conspiracy Beliefs Scale (ICBS) in confirmatory factor analysis.

## Data Availability

The dataset supporting this study’s findings is not openly available and is available from the corresponding author upon reasonable request.
