# Supplementary material for: Vaccination Readiness and Conspiracy Beliefs to Virus‐Based Diseases: A Validation Study
Source: Can J Infect Dis Med Microbiol. 2026 Aug 10;2026:9100219. doi: 10.1155/cjid/9100219 (PMC13455526; doi:10.1155/cjid/9100219)
Supplement: Supplementary file 1 [file CJID-2026-9100219-s001.docx]

**Supplementary materials**

**Table S1.** Factor loading of the COVID-19 Vaccination Readiness Scale (CVRS) and the Influenza Vaccination Readiness Scale (IVRS) in confirmatory factor analysis

| Factor | Item | Factor loading | | | |
| --- | --- | --- | --- | --- | --- |
|  |  | CVRS | | IVRS | |
|  |  | General | Specific | General | Specific |
| **Confidence** | 1. COVID-19/Influenza vaccination side effects occur rarely and are not severe for me. | 0.356 | 0.457 | 0.350 | 0.425 |
|  | 2. Political decisions about COVID-19/influenza vaccinations are scientifically grounded. | 0.711 | 0.633 | 0.734 | 0.760 |
|  | 3. I am convinced the appropriate authorities do only allow effective and safe COVID-19/influenza vaccines. | 0.690 | 0.675 | 0.738 | 0.761 |
| **Complacency**  **(R)** | 4. I do not need COVID-19/influenza vaccinations because infectious diseases do not hit me hard. (R) | 0.167 | 0.816 | 0.134 | 0.841 |
|  | 5. COVID-19/influenza vaccination is unnecessary for me because I rarely get ill anyway. (R) | 0.282 | 0.677 | 0.186 | 0.763 |
|  | 6. I get COVID-19/influenza vaccinated because it is too risky to get infected. | 0.768 | -0.176 | 0.766 | -0.077 |
| **Constraints**  **(R)** | 7. I make sure to receive the COVID-19/influenza vaccinations in good time. | 0.672 | 0.386 | 0.766 | 0.163 |
|  | 8. COVID-19/influenza vaccinations are so important to me that I prioritize getting vaccinated over other things. | 0.563 | 0.532 | 0.637 | 0.274 |
|  | 9. I sometimes miss out on COVID-19/influenza vaccinations because vaccination is bothersome. (R) | 0.192 | -0.159 | 0.587 | -0.465 |
| **Calculation**  **(R)** | 10. I get COVID-19/influenza vaccinated when I do not see disadvantages for me. (R) | -0.111 | 0.538 | -0.232 | 0.722 |
|  | 11. I only get COVID-19/influenza vaccinated when the benefits clearly outweigh the risks. (R) | -0.085 | 0.817 | -0.231 | 0.947 |
|  | 12. For each COVID-19/influenza vaccine, I carefully consider whether I need it. (R) | -0.151 | 0.546 | -0.303 | 0.455 |
| **Collective responsibility** | 13. I get COVID-19/influenza vaccinated because protecting vulnerable risk groups is important to me. | 0.467 | 0.521 | 0.469 | 0.583 |
|  | 14. I see COVID-19/influenza vaccination as a collective task against the spread of diseases. | 0.534 | 0.430 | 0.542 | 0.501 |
|  | 15. I get COVID-19/influenza vaccinated because I am thereby protecting other people. | 0.494 | 0.510 | 0.506 | 0.530 |
| **Compliance** | 16. It should be possible to exclude people from public activities (e.g., concerts) when they are not vaccinated against COVID-19/influenza. | 0.332 | 0.700 | 0.079 | 0.898 |
|  | 17. The health authorities should use all possible means to achieve high COVID-19/influenza vaccination rates. | 0.644 | 0.355 | 0.529 | 0.257 |
|  | 18. It should be possible to sanction people who do not follow the COVID-19/influenza vaccination recommendations by health authorities. | 0.307 | 0.820 | 0.082 | 0.810 |
| **Conspiracy**  **(R)** | 19. COVID-19/influenza vaccinations cause diseases and allergies that are more serious than the diseases they ought to protect from. (R) | 0.462 | 0.429 | 0.366 | 0.590 |
|  | 20. Health authorities knuckle under to the power and influence of pharmaceutical companies. (R) | 0.391 | 0.725 | 0.414 | 0.727 |
|  | 21. COVID-19/influenza vaccinations contain chemicals in toxic doses. (R) | 0.453 | 0.540 | 0.409 | 0.711 |

*Note*. Items with (R) indicate reverse-coded; factors with (R) indicate a reverse direction (e.g., higher score in Complacency means lower level of Complacency). CVRS, the COVID-19 Vaccination Readiness Scale; IVRS, the Influenza Vaccination Readiness Scale.

**Table S2.** Factor loading of the COVID-19 Conspiracy Beliefs Scale (CCBS) and the Influenza Conspiracy Beliefs Scale (ICBS) in confirmatory factor analysis.

| Factor | Item | Factor loading | |
| --- | --- | --- | --- |
|  |  | CCBS | ICBS |
| **Concealed facts and irrational suspicion** | 1. In my opinion, COVID-19/influenza does not exist. | 0.497 | 0.505 |
|  | 2. In my opinion, COVID-19/influenza is a myth that forces people to vaccinate. | 0.608 | 0.597 |
|  | 3. I think the waves emitted by 5G or 4.5G technology allow COVID-19/influenza to spread to wider areas. | 0.710 | 0.744 |
|  | 4. In my opinion, COVID-19/influenza vaccines were created to sterilize people in the long term. | 0.667 | 0.741 |
|  | 5. In my opinion, COVID-19/influenza was deliberately created to control people and manage them with microchips. | 0.746 | 0.735 |
|  | 6. The truth about the relationship between COVID-19/influenza and 5G or 4.5G radiation waves is hidden from the public. | 0.786 | 0.782 |
|  | 7. In my opinion, mRNA vaccines were produced to alter human DNA. | 0.766 | 0.746 |
| **Global control** | 8. COVID-19/Influenza is virus deliberately created by global powers aiming to reduce the world population. | 0.843 | 0.846 |
|  | 9. COVID-19/influenza is developed by states as part of a biological weapons program. | 0.644 | 0.841 |
|  | 10. I think global forces invented COVID-19/influenza to vaccinate the entire population. | 0.849 | 0.840 |
|  | 11. In my opinion, COVID-19/influenza is produced as virus that does not harm children and young people to reduce the older population in the world. | 0.753 | 0.765 |
|  | 12. I think that there are some people behind the spread of COVID-19/influenza all over the world. | 0.806 | 0.859 |
|  | 13. I think that governments are deliberately not coming up with cure for COVID-19/influenza. | 0.696 | 0.668 |

*Note.* CCBS, the COVID-19 Conspiracy Beliefs Scale; ICBS, the Influenza Conspiracy Beliefs Scale.
